# Temporal evolution of HIV sero-discordancy patterns among stable couples in sub-Saharan Africa

**DOI:** 10.1371/journal.pone.0196613

**Published:** 2018-04-30

**Authors:** Susanne F. Awad, Hiam Chemaitelly, Laith J. Abu-Raddad

**Affiliations:** 1 Infectious Disease Epidemiology Group, Weill Cornell Medicine-Qatar, Cornell University, Qatar Foundation - Education City, Doha, Qatar; 2 Population Health Research Institute, St George’s, University of London, London, United Kingdom; 3 Department of Healthcare Policy and Research, Weill Cornell Medicine, Cornell University, New York, New York, United States of America; The Ohio State University, UNITED STATES

## Abstract

**Introduction:**

Objective was to examine the temporal variation of HIV sero-discordancy in select representative countries (Kenya, Lesotho, Mali, Niger, Tanzania, and Zimbabwe) in sub-Saharan Africa at different HIV epidemic scales. A sero-discordant couple is defined as a stable couple (SC) in which one partner is HIV-positive while the other is HIV-negative.

**Methods:**

A deterministic compartmental mathematical model was constructed to describe HIV transmission dynamics. The model was pair-based, that is explicitly modeling formation of SCs and infection dynamics in both SCs and in single individuals. The model accommodated for different forms of infection statuses in SCs. Using population-based nationally-representative epidemiologic and demographic input data, historical (1980–2014) and future (2015–2030) trends of sero-discordancy and other demographic and epidemiologic indicators were projected throughout HIV epidemic phases.

**Results:**

As the epidemics emerged, about 90% of SCs affected by HIV were sero-discordant. This proportion declined to 45%-88% at epidemic peak and stabilized as the epidemics started their natural decline. The largest reductions in sero-discordancy were in high HIV-prevalence countries. As the epidemics further declined with antiretroviral therapy (ART) scale-up, the proportion of sero-discordant couples among HIV-affected couples was projected to increase to 70%-92% by 2030. The proportion of sero-discordant couples among all SCs increased as the epidemics emerged and evolved, then peaked at 2%-20% as the epidemics peaked, and then declined as the epidemics declined to reach 0.3%-16% by 2030.

**Conclusions:**

Sero-discordancy patterns varied with the evolution of the epidemics, and were affected by both epidemic phase and scale. The largest variations were found in high HIV-prevalence countries. The fraction of stable couples that are sero-discordant, as opposed to being sero-concordant positive, was projected to increase with ART scale-up and further HIV incidence decline over the coming two decades. These findings inform strategic planning and resource allocation for interventions among sero-discordant couples.

## Introduction

Between 35 and 76% of the adult population in sub-Saharan Africa (SSA) live in stable sexual couples (SCs) [1–4]. Of these couples, 1 to 45% are affected by HIV [2], among whom approximately one in every two in high HIV-prevalence countries is HIV sero-discordant—that is, one partner is HIV-positive while the other is HIV-negative [2–4]. HIV incidence in sero-discordant couples (SDCs) contributes about 30% of all new HIV infections, thus sustaining HIV epidemics in SSA [2, 5–7], and leading to SDCs becoming a priority population for HIV prevention efforts with a number of demonstrated efficacious interventions among them [5, 8–10].

Recent research on HIV epidemiology among SCs in SSA provided a mapping of HIV sero-discordancy patterns [2], and delineated the sources of HIV infection among SCs and their contribution to incidence [4, 7, 11]. These studies have also demonstrated substantial variations across countries in the patterns of sero-discordancy [2, 4, 7, 11]. The reasons behind this variability remain poorly understood. Several potential explanations have been suggested [12, 13]. One of these is HIV epidemic phase (i.e. emergence phase, epidemic-peak phase, and “natural” epidemic decline phase), and epidemic temporal evolution (that is how the epidemic has changed over time) considering the rapid changes in incidence and prevalence in much of SSA over the last two decades [14–16]. Future projections of sero-discordancy over the coming decades, and of the role of SDCs in the epidemic, are also uncertain, given the expected changes in incidence and prevalence with the scale-up of interventions—in particular antiretroviral therapy (ART).

Delineating the historical variation of sero-discordancy with time in Africa’s epidemics and future projections are therefore critical to inform HIV policy and programming, and the role that prevention interventions can play for SDCs. Accordingly, we aimed to examine the temporal variation of sero-discordancy patterns in six representative SSA countries (at different HIV epidemic scales) using a dynamic pair-based mathematical model—that is a model that explicitly simulates formation of SCs and infection dynamics in both SCs and in single individuals, and accommodates for the variability in sexual risk behavior and mixing in the population. Specifically, we explored historical trends (between 1980 and 2014) and forecasted future temporal variation (between 2015 and 2030) of key sero-discordancy indicators.

The overarching aim of our study was to inform our understanding of HIV epidemiology among SCs, and how the changes in incidence and prevalence are likely to affect epidemiological patterns. Furthermore, we aspired to explore the extent to which the variation in sero- discordancy patterns could be explained by epidemics dynamics.

## Methods

We constructed a deterministic compartmental mathematical model of HIV transmission dynamics based on extension of an earlier model [12]. The model was pair-based simulating explicitly SC formation and infection transmission in both SCs and in single individuals. The model was parameterized using nationally-representative and population-based demographic and epidemiological measures derived from sources such as the Demographic and Health Surveys (DHS) [17]. All data were already fully anonymized before accessing them. The model was coded and analyzed in MATLAB R2015a [18]. The MATLAB codes can be obtained by contacting the authors.

### Mathematical model and parametrization

**Fig 1:**
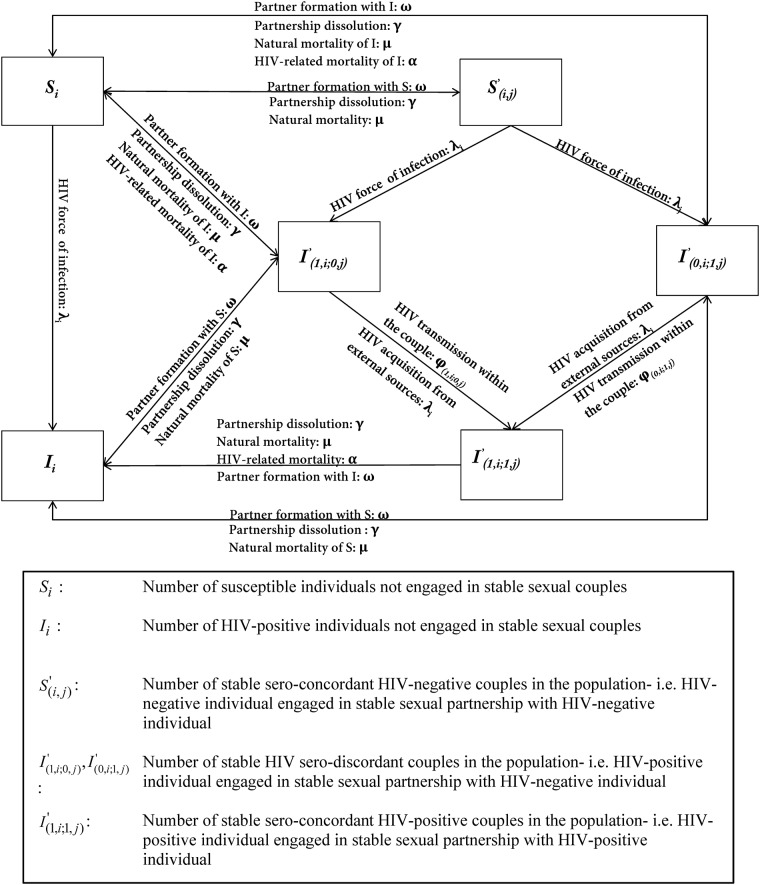
Flow-chart depicting the basic structure of the used pair-based HIV model.

The model consisted of a system of coupled nonlinear ordinary differential equations representing the movement of individuals between different forms of sexual partnerships and HIV infection statuses (Fig 1). Model compartments stratify the population based on HIV status, sexual risk group, engagement in a SC, state of sero-discordancy or sero-concordancy within the SC, and ART treatment status.

The sexual contact structure accommodates heterogeneity in sexual risk behavior and different forms of mixing between risk groups. The population in reproductive age was stratified into 10 sexual risk groups representing the variable risk to HIV exposure [19]. This number of risk groups was included to represent the diversity of sexual risk behavior that exists in a given population, starting from the lowest level (risk group 1) to the highest level (risk group 10) of sexual risk behavior. For example, risk group 1 represented the low risk general population that has the lowest risk of acquiring HIV in a given population. Meanwhile, risk group 10 represented the high risk populations that have the highest risk of acquiring HIV, such as female sex workers and their male clients.

The proportion of the population in each risk group was informed by data from SSA on the distribution of the number of sexual partners over the past year [20]. The population average level of sexual risk behavior was assumed to vary during the epidemic, based on evidence suggesting substantial declines in the risk of HIV exposure across SSA [16].

We described the mixing between the different sexual risk groups through a mixing matrix. The matrix incorporated both an assortative component where partners were chosen preferentially from within the same risk group (i.e. a low risk individual choosing a sexual partner also with low risk), and a proportionate component where partners were chosen with no preferential bias based on risk group.

**Fig 2:**
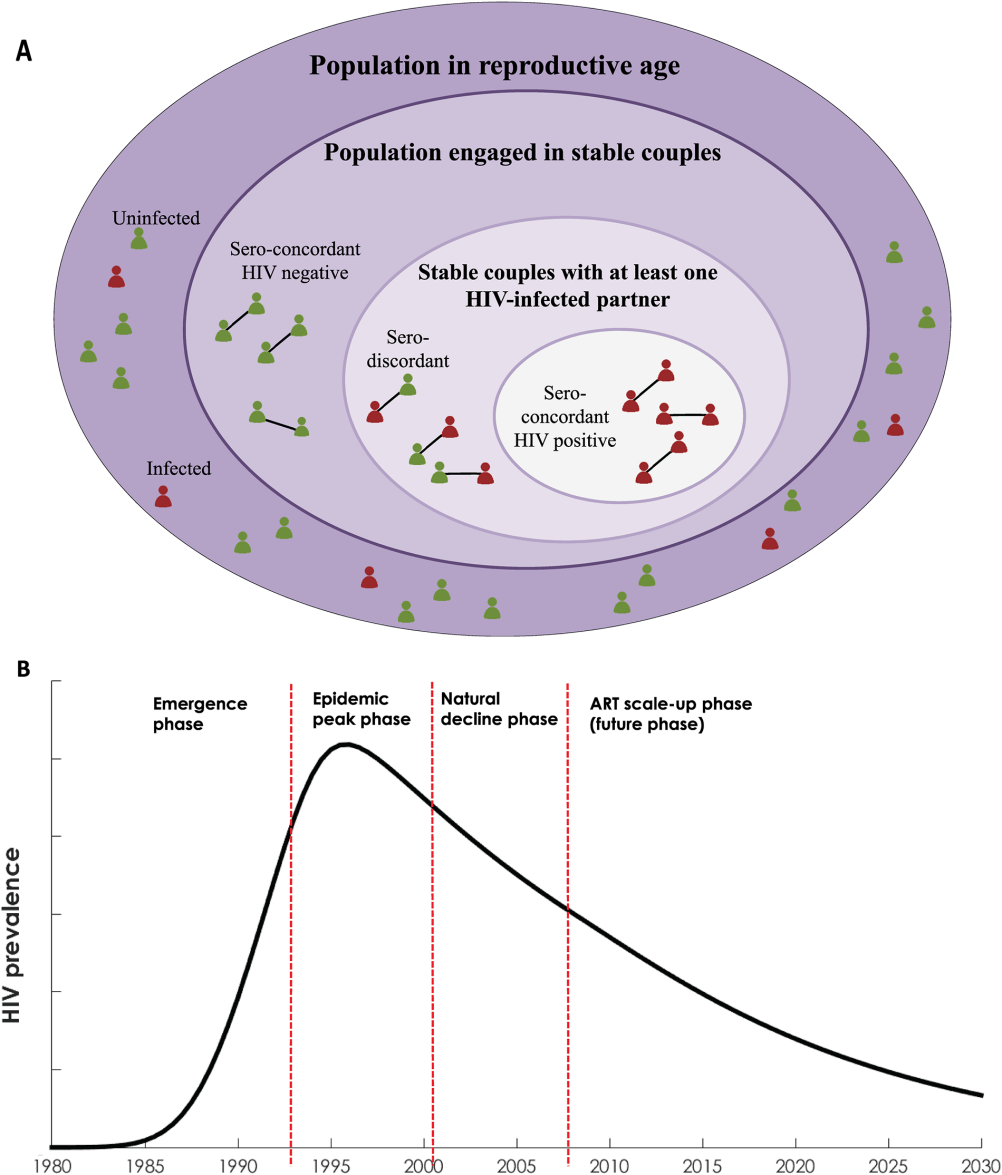
(A) Conceptual diagram illutrating the population in reproductive age by partnership status: not engaged in a stable couple (singles), sero-concordant HIV-negative stable couple, HIV sero-discordant stable couple, and sero-concordant HIV-positive stable couple. (B) Characterization of the four generic phases of the HIV epidemics in sub-Saharan Africa. The sero-discordancy patterns in each country were described and analyzed within the context of these phases of each country’s epidemic.

Stable couples were divided into SDCs, sero-concordant HIV-negative couples (both partners are sero-negative), and sero-concordant HIV-positive couples (both partners are sero-positive; Fig 2A). ART scale-up was assumed to start in 2008 and increase at a fixed rate to reach the country-specific ART coverage in 2014 [21]. The same fixed scale-up rate was assumed to continue after 2014. ART was assumed to reduce the risk of HIV transmission by 96% based on randomized clinical trial and observational data [10, 22], and to slow the rate of disease progression by 67% consistent with assumptions of the Joint United Nations Programme on HIV/AIDS (UNAIDS) SPECTRUM model [23, 24].

Our model was parameterized using current empirical HIV natural history and epidemiology data from SSA, as well as through model fitting for some of the parameters. Parameter values are described in S1 Table along with their specific sources. These model parameters were an outcome and distillation of a process through a series of mathematical modeling studies investigating different aspects of HIV transmission dynamics in SSA [2, 12, 16, 20, 25–27]. HIV prevalence and sero-discordancy data were extracted from DHS databases [17]. HIV prevalence historical trend data were provided through UNAIDS estimations [28].

Further details on the mathematical model, its structure, and its parametrization can be found in S1 Text.

### Model fitting

Model predictions were fitted to the country-specific HIV prevalence time series (1990–2014), and seven country-specific demographic and epidemiologic indicators for each country. The indicators were: population prevalence of SCs (i.e. marriage rate; *P*_*couples*_), population HIV prevalence (*P*), proportion of couples affected by HIV among all SCs (*P*_*pos*_), proportion of SDCs among all SCs with at least one HIV-infected individual in the couple (*P*_*discord*_), proportion of SDCs among all SCs in the population (*P*_*all*_), proportion of individuals engaged in SDCs among the entire population in reproductive age (*I*_*all*_), and proportion of HIV-infected individuals engaged in SDCs among all HIV-infected individuals (*I*_*pos*_).

The empirical measures of these indicators were derived from the DHS databases [17], and then estimated and fitted through the model as described in the S1 Text. A summary of the definitions, interpretations, and estimations of these key sero-discordancy indicators can be found in Table 1, and further details can be found in Section D of S1 Text and in Chemaitelly et al [2].

**Table 1:** Population-level demographic and epidemiological indicators relating to HIV sero-discordancy by epidemic phase for six countries in sub-Saharan Africa representing different HIV epidemic scales: Low HIV prevalence (Niger and Mali), intermediate HIV prevalence (Tanzania and Kenya), and high HIV prevalence (Zimbabwe and Lesotho).

Measure	Definition	Interpretation	Estimation	HIV epidemic phase			
				Emerging phase	Epidemic peak	Natural epidemic decline phase	Declining epidemic with ART^§^ scale-up (by 2030)
*P*_*couples*_	Population prevalence of SCs*	Measures the level of engagement of individuals in SCs in the population^#^	$\frac{\text{number}\;\text{of}\;\text{individuals}\;\text{in}\;\text{SCs}\;}{\text{total}\;\text{number}\;\text{of}\;\text{individuals}\;}$	51%-84%-	49%-84%	49%-84%	51%-84%
*P*	HIV prevalence in the population	Measures the level of HIV-infected individuals in the population^#^	$\frac{\text{total}\;\text{number}\;\text{of}\;\text{HIV}-\text{infected}\;\text{individuals}}{\text{total}\;\text{number}\;\text{of}\;\text{individuals}\;}$	0.0%-27%	1%-30%	0.5%-28%	0.2%-13%
*P*_*pos*_	Proportion of couples affected by HIV out of all SCs	Measures the proportion of SCs affected by HIV among all SCs in the population^#^	$\frac{\text{number}\;\text{of}\;\text{SCs}\;\text{affected}\;\text{by}\;\text{HIV}}{\text{total}\;\text{number}\;\text{of}\;\text{SCs}}$	0.0%-40%	2%-41%	1%-37%	0.3%-22%
*I*_*pos*_	Proportion of all HIV-infected individuals engaged in a stable SDCs^€^	Measures the level of engagement of HIV-infected individuals in SDCs	$\frac{\text{number}\;\text{of}\;\text{HIV-infected}\;\text{individuals}\;\text{in}\;\text{SDCs}}{\text{total}\;\text{number}\;\text{of}\;\text{HIV}-\text{infected}\;\text{individuals}}$	21%-67%	15%-63%	17%-60%	31%-68%
*P*_*all*_	Proportion of SDCs among all SCs in the population	Measures the level of sero-discordancy among all SCs in the population^#^	$\frac{\text{number}\;\text{of}\;\text{SDCs}}{\text{total}\;\text{number}\;\text{of}\;\text{SCs}}$	0.0%-20%	2%-21%	1%-17%	0.3%-16%
*I*_*all*_	Proportion of individuals engaged in SDCs in the population	Measures the abundance of individuals who are engaged in SDCs in the population^#^	$\frac{\text{number}\;\text{of}\;\text{individuals}\;\text{in}\;\text{SDCs}}{\text{total}\;\text{number}\;\text{of}\;\text{individuals}\;}$	0.0%-11%	2%-12%	1%-9%	0.2–8%
*P*_*discord*_	Proportion of SDCs among all SCs with at least one HIV-infected individual in the couple	Measures the proportion of SCs affected by HIV where the uninfected partner is at risk of acquiring HIV from the infected partner	$\frac{\text{number}\;\text{of}\;\text{SDCs}}{\begin{array}{l} \text{total}\;\text{number}\;\text{of}\;\text{SCs}\;\text{with}\;\text{at}\;\text{least}\;\text{one}\;\\ \text{HIV}-\text{infected}\;\text{individual}\;\text{in}\;\text{the}\;\text{couple} \end{array}}$	54%-93%	45%-88%	45%-87%	70%-92%

*SC: stable couple.

^#^ population: the entire population in reproductive age (15–49 years old).

^§^ART: antiretroviral therapy.

^€^SDC: stable HIV sero-discordant couple.

### Plan of analysis

Six countries in SSA representing different HIV epidemic scales were included in our analysis: low HIV prevalence (Niger and Mali), intermediate HIV prevalence (Tanzania and Kenya), and high HIV prevalence (Zimbabwe and Lesotho). Country choices were informed by the availability of sufficient data to characterize the trend in HIV prevalence at the national level, and by the availability of more than one DHS HIV serological biomarker survey. These countries were also chosen to represent a diversity of epidemic levels (i.e. low, intermediate, and high). The analysis was conducted at the country level, rather than at the regional level, to utilize the available country-level nationally-representative population-based DHS data. Consequently, sero-discordancy was analyzed in Kenya (DHS 2003 and 2008–09), Lesotho (DHS 2004 and 2009), Mali (DHS 2006 and 2012–13), Niger (DHS 2006 and 2012), Tanzania (DHS 2003–04, 2007–08, and 2012), and Zimbabwe (DHS 2005–06 and 2011).

The HIV epidemic in each country was divided temporally into four phases (Fig 2B): emergence phase as the epidemic started its growth, epidemic-peak phase as the epidemic reached its highest HIV prevalence, “natural” decline phase as the epidemic started to decline following the peak level, and ART scale-up phase (future phase) as the epidemic further declined with high coverage of ART. The natural decline phase was labeled as “natural” to distinguish it from the subsequent decline with ART scale-up and its high coverage. The exact drivers of the declines in epidemics over the last two decades or so remain not well-understood [16], and possibly reflect behavioral changes and natural epidemic dynamics [16]. Sero-discordancy patterns were described and analyzed within the context of these four epidemic phases.

Using the best-fit parameters of the pair-based model for each country, we assessed the historical trends in the following indicators: *P*, *P*_*couples*_, *P*_*pos*_, *P*_*discord*_, *I*_*pos*_, *P*_*all*_, and *I*_*all*_ between 1980 and 2014, the timeframe in which the epidemics emerged, peaked, and naturally declined. We also examined future trends in these indicators between 2015 and 2030, the timeframe in which ART had substantial coverage and the epidemics were predicted to decline further.

## Results

The discordancy measures that were included in the fitting are listed in S3 Table, while the fitted parameters for each country are listed in S2 Table. The mathematical model fitted HIV prevalence trends and the rest of the indicators. The various sero-discordancy and other demographic and epidemiologic indicators showed different variations with epidemic phase, as summarized in Table 1. The prevalence of SCs (*P*_*couples*_) was stable between 1980 and 2030 for all countries (S1 Fig). *P*_*couples*_ ranged between 51.3% (Lesotho) and 84.4% (Mali).

The proportion of SCs affected by HIV among all SCs (*P*_*pos*_) varied over time (S3 Fig), closely following the variation of HIV prevalence in each country (S2 Fig). Early in the epidemics, *P*_*pos*_ emerged from 0.0% to between 2% (Niger) and 40% (Zimbabwe). As the epidemics reached their peak, *P*_*pos*_ also peaked at between 2% (Niger) and 41% (Zimbabwe). As the epidemics started to naturally decline, *P*_*pos*_ declined, following the declines in HIV prevalence. By 2030, with ART scale-up, between 0.3% (Niger) and 22% (Lesotho) of SCs would be affected by HIV (S3 Fig).

**Fig 3:**
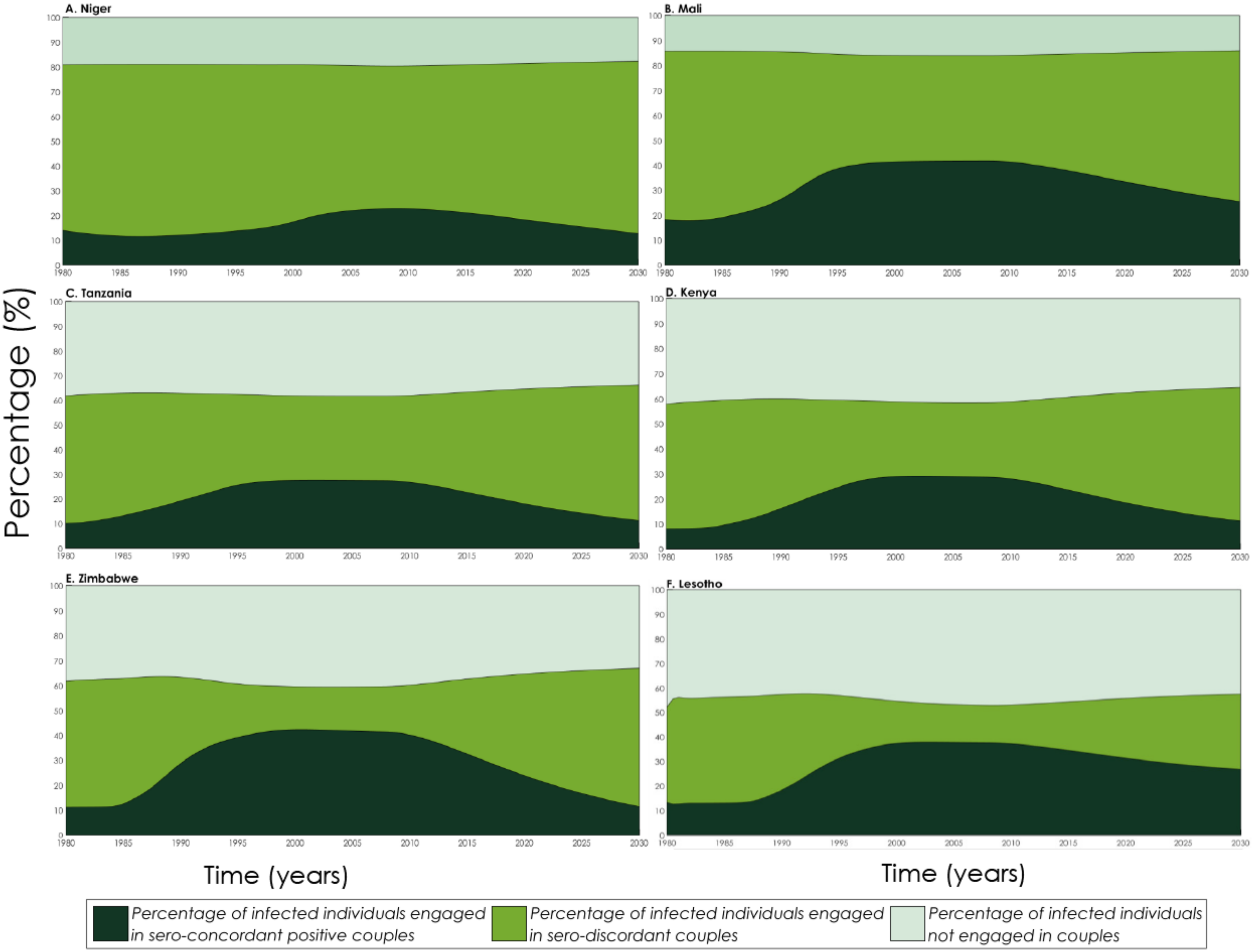
Model predicted proportion of HIV-infected individuals by partnership status: Not engaged in stable couple, sero-discordant stable couple, and sero-concordant positive stable couple. Countries are shown in order of increasing HIV prevalence. (A) Niger, (B) Mali, (C) Tanzania, (D) Kenya, (E) Zimbabwe, and (F) Lesotho.

Fig 3 shows the proportion of HIV-infected individuals in each partnership status (not engaged in couples (singles), SDCs, and sero-concordant positive couples). The proportion of infected individuals not engaged in couples was largely stable throughout the epidemics in all countries. However, this proportion was lower in countries with low HIV prevalence, as marriage rates were higher (S1 Fig). Only 20% of infected individuals were not engaged in couples in Niger (low HIV-prevalence country) compared to 40% in Lesotho (high HIV-prevalence country).

The proportion of HIV-infected individuals in SDCs (*I*_*pos*_) varied with time throughout the epidemics (Fig 3 and S4 Fig). In the epidemics’ emerging phase, between 21% (Zimbabwe) and 67% (Mali) of infected individuals were in SDCs. As the epidemics evolved and peaked, this proportion declined to reach a minimum of between 15% (Lesotho) and 57% (Niger). This proportion remained stable at this level at the epidemics’ natural decline phase. As the epidemics declined further with ART scale-up, this proportion increased again to reach between 31% (Lesotho) and 68% (Niger) by 2030.

The proportion of HIV-infected individuals that are in sero-concordant positive couples varied also with time throughout the epidemics (Fig 3). In the epidemics’ emerging phase, between 9% (Kenya) and 36% (Zimbabwe) of infected individuals were in sero-concordant positive couples. This proportion increased to between 17% (Niger) and 41% (Zimbabwe) as the epidemics evolved and peaked, and remained then stable in the epidemics’ natural decline phase. As the epidemics declined further with ART scale-up, this proportion declined to reach between 11% (Tanzania) and 27% (Lesotho) by 2030.

**Fig 4:**
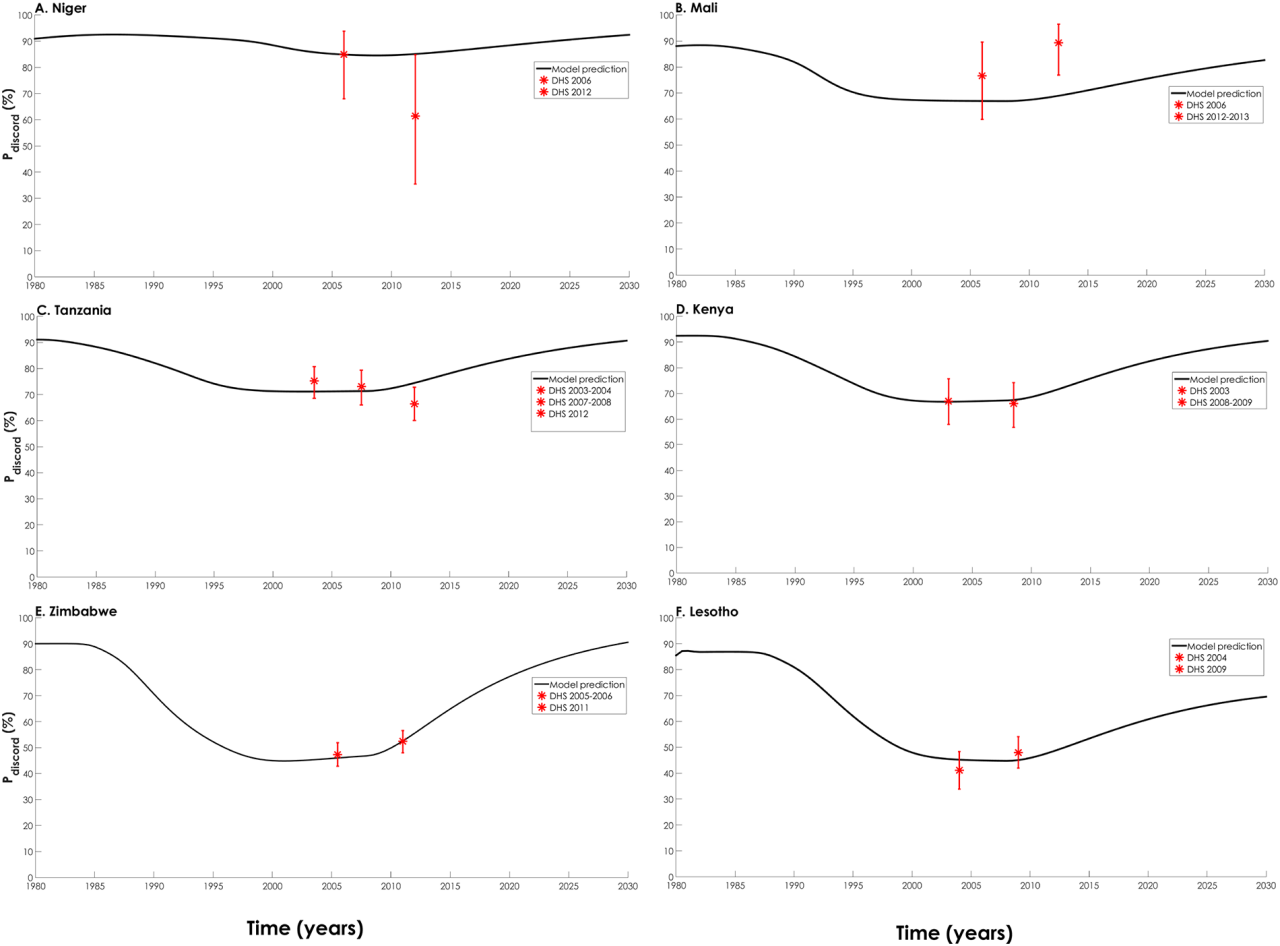
Model predicted proportion of stable HIV sero-discordant couples among all stable couples with at least one HIV-infected individual in the couple (*P*_*discord*_) for six representative countries in sub-Saharan Africa. Countries are shown in order of increasing HIV prevalence: (A) Niger, (B) Mali, (C) Tanzania, (D) Kenya, (E) Zimbabwe, and (F) Lesotho. The black lines show model predictions while the red asterisks show Demographic and Health Surveys data points and their 95% confidence interval [17].

As the epidemics emerged, the proportion of SDCs among all SCs with at least one HIV-infected individual in the couple (*P*_*discord*_), the most important indicator of sero-discordancy, started at about 90% (Fig 4). As the epidemics evolved and peaked, *P*_*discord*_ ranged between 45% (Zimbabwe) and 88% (Niger), and then stabilized during the epidemics’ natural decline phase. As the epidemics declined further with ART scale-up, *P*_*discord*_ increased to between 70% (Lesotho) and 92% (Niger) by 2030. The variation in *P*_*discord*_ with epidemic phase was more pronounced with higher HIV prevalence. For example, as the epidemics evolved, *P*_*discord*_ in Zimbabwe, a high HIV-prevalence country, declined from 90% to 45% (Fig 4E), while *P*_*discord*_ in Niger, a low HIV-prevalence country, declined slightly from 91% to 85% (Fig 4A).

The proportion of SDCs among all SCs (*P*_*all*_) was low early in the epidemics and increased as the epidemics expanded, peaking around the same year that HIV prevalence peaked (S5 Fig). The peak value of *P*_*all*_ ranged between 2% (Niger) and 21% (Zimbabwe). As the epidemics started their natural decline, *P*_*all*_ declined, following also the decline in HIV prevalence (S2 Fig). As the epidemics declined further with ART scale-up, *P*_*all*_ increased slightly in high HIV-prevalence countries (Panels E and F in S5 Fig), and then declined again with further decline in HIV prevalence. By 2030, *P*_*all*_ ranged between 0.3% (Niger) and 16% (Lesotho).

The proportion of individuals engaged in SDCs among the entire population in reproductive age (*I*_*all*_; S6 Fig) closely followed the trends of *P*_*all*_ (S5 Fig) and HIV prevalence (S2 Fig). In the epidemics’ emerging phase, *I*_*all*_ emerged from 0.0% to between 1% (Niger) and 11% (Zimbabwe). As the epidemics evolved and peaked, *I*_*all*_ peaked at 2% (Niger) to 12% (Zimbabwe). As the epidemics started their natural decline, *I*_*all*_ declined, following also the decline in *P*_*all*_ and HIV prevalence. As the epidemics declined further with ART scale-up, *I*_*all*_ increased slightly in high HIV-prevalence countries (Panels E and F in S6 Fig), and then declined again with further decline in HIV prevalence. By 2030, *I*_*all*_ was projected to range between 0.2% (Niger) and 8% (Lesotho).

S7 Fig highlights the impact of ART scale-up on *P*_*discord*_. The figure shows *P*_*discord*_ with ART scale-up compared to a counter-factual scenario of no-ART scale up, in Kenya as an illustrative example. The temporal variation of *P*_*discord*_ was sensitive to ART coverage, and *P*_*discord*_ increased steadily as ART was scaled up. The temporal variation of *I*_*pos*_ was also sensitive to ART coverage, while the impact of ART scale-up was rather limited on *P*_*pos*_, *P*_*all*_, and *I*_*all*_ (not shown). While the impact of ART scale-up is shown here only for Kenya, similar patterns were observed in the other countries.

## Discussion

The patterns of sero-discordancy in SSA and the role of SDCs in the epidemics have been subject of debate [29–31]. The debate was centered on data derived from cross-sectional surveys at specific points of time, mostly post the year 2000. We presented here, however, a comprehensive analysis of different sero-discordancy indicators throughout the epidemics’ phases, and projected the indicators’ variation into the future as the epidemics further decline with ART scale-up.

Our results indicated that sero-discordancy patterns varied with the epidemics’ evolution, reflecting rich infection dynamics. Our findings demonstrated that cross-sectional measures of sero-discordancy reflect only snapshots that are not necessarily representative, and that these snapshots may lead to misinterpretations about the role of SCs in HIV epidemiology. Proper understanding of sero-discordancy epidemiology cannot be accomplished without factoring the time dimension. This comes in contrast with the spatial dimension, which turned out to contribute minimally to understanding sero-discordancy epidemiology and its patterns [32] These findings corroborate earlier studies suggesting that HIV epidemiology among SCs appears to be a predictable “spill-over” effect of the “core” HIV dynamics in the population [2, 4, 7, 11, 33], and accordingly is affected by change in epidemic phase.

One of the consistently observed patterns in cross-sectional data is that the fraction of SCs affected by HIV that are sero-discordant as opposed to being sero-concordant positive (*P*_*discord*_), is much higher in low HIV-prevalence than in high HIV-prevalence countries [2] Our results demonstrated that this pattern is largely temporal in nature, and emerged only because these cross-sectional data were collected when *P*_*discord*_ was at its nadir as the epidemics started their natural decline (Fig 4).

A variation and compensation pattern, between sero-discordant versus sero-concordant positive SCs, was evident throughout the epidemics (Fig 3). Historically, even in high HIV-prevalence countries, *P*_*discord*_ was very high (around 90%) early in the epidemics when HIV transmission was focused primarily in high-risk populations. At this time, with HIV incidence among those aged 15–24 being substantial, many young individuals acquired the infection through premarital sex, but then married negative partners and formed new SDCs. Others, who were already in SCs, brought the infection into the partnership through extramarital sex, thereby also forming new SDCs. As the epidemics expanded into the general population and reached their peaks (in the 1990s), many of these SDCs were becoming sero-concordant positive as the infected index partners passed the infection to their spouses.

As HIV incidence rate started to decline, existing SDCs became sero-concordant positive at a faster rate than partnerships became SDCs—the risk of acquiring the infection through extramarital sex was declining, and there were less young individuals being infected through premarital sex to marry and form new SDCs. With this imbalance in more SDCs becoming sero-concordant positive, and less SDCs being formed, half of SCs affected by HIV in high HIV-prevalence countries became sero-concordant positive (Fig 3). As we move into the future with high ART coverage over the coming two decades, many of the sero-concordant positive SCs would have been dissolved due to disease mortality of one or both of the partners, or because of partnership dissolution. With ART treatment also, infected index partners in SDCs are less likely to transmit the infection to their spouses. These trends will result in the vast majority of partnerships affected by HIV being sero-discordant, in both high and low HIV-prevalence countries. The ART era will bring *P*_*discord*_ back to its high historical levels when the epidemics emerged (Fig 4).

The variations in sero-discordancy indicators were found to be more pronounced in high HIV-prevalence countries, given the larger variation in epidemic scale across the different epidemic phases (Figs 3 and 4 and S3–S6 Figs). Our results also indicated that at epidemic peak, very large fraction of SCs were affected by HIV in the high HIV-prevalence countries (S3 Fig). As much as 40% of SCs in Lesotho and Zimbabwe were affected by HIV, with one or both partners being infected, highlighting the severity of these epidemics at that time even in the general population of married individuals. As the epidemics further decline with ART scale-up over the next two decades, fewer couples will be affected by HIV, but still 10–20% of couples in the high HIV-prevalence countries will remain affected.

Our results further suggested that the prevalence of SCs (marriage) has been largely stable despite AIDS mortality, even in high HIV-prevalence countries (S2 Fig). Moreover, the fraction of infected individuals who are not part of a SC has been also overall stable, and was higher with higher HIV prevalence (Fig 3). As much as 40% of infected individuals are not in SCs in Lesotho and Zimbabwe. This outcome is a consequence of the lower average age at infection in high HIV-prevalence countries—many young individuals acquire the infection before getting married. Disease mortality also contributed to this outcome, though modestly, as a fraction of SCs were dissolved due to death of one of the partners.

Though we used a complex mathematical model structure to capture the complexity of HIV dynamics among SCs, our predictions may depend on the type of mathematical model used in the analyses. Our analyses are also limited by the quality and precision of available data. DHS data are quality population-based and nationally-representative data, but have a number of inherent limitations [2, 12]. Despite these potential sources of limitations, our model fitted measured sero-discordancy and other indicators across the countries (Fig 4 and S1–S6 Figs).

## Conclusions

SDCs are a key population for HIV prevention, and several interventions with proven efficacies can benefit them. However, sero-discordancy patterns varied with time with the evolution of the epidemics, and were affected by both epidemic phase and scale. The largest variations in sero-discordancy were found in high HIV-prevalence countries. The fraction of SCs that are sero-discordant, as opposed to being sero-concordant positive, was projected to increase with ART scale-up and further HIV incidence decline over the coming two decades. These findings inform strategic planning and resource allocation for intervention programming among SDCs.

## Supporting information

S1 Text The mathematical model. (DOCX)

S1 Table Model assumptions in terms of parameter values. (DOCX)

S2 Table Estimated parameter values for the six countries included in our study. (DOCX)

S3 Table Empirical values and 95 confidence interval (CI) of HIV prevalence and the key HIV sero-discordancy measures for six representative countries in sub-Saharan Africa as estimated from the Demographic Health Surveys (DHS) databases. (DOCX)

S1 Fig Model predicted population prevalence of stable couples (*P*_*couples*_) for six representative countries in sub-Saharan Africa. (DOCX)

S2 Fig Model predicted HIV prevalence among the population in reproductive age for six representative countries in sub-Saharan Africa. (DOCX)

S3 Fig Model predicted proportion of couples affected by HIV out of all stable couples (*P*_*pos*_) for six representative countries in sub-Saharan Africa. (DOCX)

S4 Fig Model predicted proportion of all HIV-infected individuals in the population that are engaged in stable HIV sero-discordant couples (*I*_*pos*_) for six representative countries in sub-Saharan Africa. (DOCX)

S5 Fig Model predicted proportion of stable HIV sero-discordant couples among all stable couples in the population (*P*_*all*_) for six representative countries in sub-Saharan Africa. (DOCX)

S6 Fig Model predicted proportion of individuals in a stable HIV sero-discordant couple among the entire population in reproductive age (*I*_*all*_) for six representative countries in sub-Saharan Africa. (DOCX)

S7 Fig Model predicted impact of antiretroviral therapy (ART) scale-up on HIV sero-discordancy. (DOCX)
