# Supplementary material for: Temporal evolution of HIV sero-discordancy patterns among stable couples in sub-Saharan Africa
Source: PLoS One. 2018 Apr 30;13(4):e0196613. doi: 10.1371/journal.pone.0196613 (PMC5927442; doi:10.1371/journal.pone.0196613)
Supplement: S1 Text — (DOCX) [file pone.0196613.s001.docx]

**S1 Text - The mathematical model**

- 1. **Model structure and equations**

A pair-based deterministic compartmental mathematical model of the dynamics of HIV transmission in a population was constructed based on extension of an earlier model [[1](#_ENREF_1)]. The model stratifies the sexually active population into compartments according to HIV status, sexual risk group, engagement in a stable couple (SC), state of sero-discordancy or sero-concordancy within the SC, and antiretroviral therapy (ART) treatment status (Fig 1 of main manuscript).

The model was expressed using sets of coupled nonlinear ordinary differential equations, each of which represents a specific risk group *i*:

To accommodate for antiretroviral therapy (ART) scale-up, we further stratified the infected population into groups according to ART status, defined with the index *ART* for individuals on ART:

To accommodate heterogeneity in sexual risk behavior, we stratified the population into ten sexual risk groups with increasing level of sexual risk behavior, defined with the index *i* or *j* ($i,j=1,2...,10$representing the low to higher risk groups).

In the above equations, $S_{i}$ is the number of susceptible individuals in the *i*-risk group in the population not engaged in stable sexual partnerships, and $I_{i}$ and $I_{i,ART}$ are the numbers of HIV-positive individuals in the *i*-risk group in the population not engaged in stable sexual couples. $S_{(i,j)}^{'}$ is the number of stable sero-concordant HIV negative couples in the population—i.e. susceptible individuals in the *i*-risk group engaged in stable sexual partnerships with susceptible individuals in the *j*-risk group. $I_{(1,i;0,j)}^{'}$, $I_{(1,i,ART;0,j)}^{'}$, $I_{(0,i;1,j)}^{'}$, and $I_{(0,i;1,j,ART)}^{'}$ represent the numbers of stable HIV sero-discordant couples in the population. That is, HIV-positive individuals in the *i*-risk group engaged in stable sexual partnerships with susceptible individuals in the *j*-risk group ($I_{(1,i;0,j)}^{'}$, $I_{(1,i,ART;0,j)}^{'}$), or HIV-positive individuals in the *j*-risk group engaged in stable sexual partnerships with susceptible individuals in the *i*-risk group ($I_{(0,i;1,j)}^{'}$,$I_{(0,i;1,j,ART)}^{'}$). $I_{(1,i;1,j)}^{'}$, $I_{(1,i,ART;1,j)}^{'}$, $I_{(1,i;1,j,ART)}^{'}$, and $I_{(1,i,ART;1,j,ART)}^{'}$ are the numbers of stable sero-concordant HIV positive couples in the population—i.e. HIV-positive individuals in the *i*-risk group engaged in stable sexual partnerships with HIV-positive individuals in the *j*-risk group. $N_{0}$ refers to the initial population size of each *i*-risk group, while $N_{s}$ refers to the population size of individuals not engaged in stable sexual partnerships for each *i*-risk group. $\mu$ is the rate at which individuals leave the population in reproductive age. The rate $\alpha$ is HIV/AIDS disease mortality rate for HIV infected not on ART, while $\alpha_{ART}$ is HIV/AIDS disease mortality rate for HIV infected on ART, respectively. $\delta_{ij}$ is the Kroenker delta (identity matrix), $\gamma$ is the partnership dissolution rate (i.e. $\frac{1}{d}$, where $d$ is the duration of stable sexual partnerships), while $\omega$ is the rate of stable partnership formation. The rate $\eta$ is the ART treatment rate. The rate is the risk of HIV transmission from the infected to the uninfected partner in a stable HIV sero-discordant couple in absence of ART, while is the risk of HIV transmission from the infected on ART to the uninfected partner in a stable HIV sero-discordant couple. The factor $r$ is the reduction in HIV transmission probability per coital act due to ART. The rate $\lambda_{i}$ is the HIV force of infection (incidence rate of infection) experienced by each susceptible population (among couples it is the incidence rate of infection from external sources for each susceptible partner in the couple). $\lambda_{i}$ is given by:

$$\begin{matrix} \lambda_{i}=\rho_{S_{i}}\left(\sum_{j=1}^{N_{risk}} t_{I_{j}\to S_{i}}G_{ij}\frac{\rho_{I(1,j)}I_{j}}{H_{j}}+\sum_{k=1}^{N_{risk}} \sum_{j=1}^{N_{risk}} \tilde{t}_{I^{'}_{(0,k;1,j)}\to S_{i}}G_{ij}\right.\frac{\rho_{I^{'}_{(0,k;1,j)}}I^{'}_{(0,k;1,j)}}{H_{j}} \\ +\sum_{k=1}^{N_{risk}} \sum_{j=1}^{N_{risk}} \tilde{t}_{I^{'}_{(1,k;0,j)}\to S_{i}}G_{ik}\frac{\rho_{I_{(1,k;0,j)}^{'}}I^{'}_{(1,k;0,j)}}{H_{k}}+\sum_{k=1}^{N_{risk}} \sum_{j=1}^{N_{risk}} \tilde{t}_{I^{'}_{(1,k;1,j)}\to S_{i}}G_{ij}\frac{\rho_{I^{'}_{(1,k;1,j)}}I^{'}_{(1,k;1,j)}}{H_{j}} \\ +\sum_{j=1}^{N_{risk}} \sum_{k=1}^{N_{risk}} \tilde{t}_{I^{'}_{(1,k;1,j)}\to S_{i}}G_{ik}\frac{\rho_{I^{'}_{(1,k;1,j)}}I^{'}_{(1,k;1,j)}}{H_{k}}+\sum_{j=1}^{N_{risk}} t_{I_{j,ART}\to S_{i}}G_{ij}\frac{\rho_{I_{j,ART}}I_{j,AR)}}{H_{j}} \\ +\sum_{k=1}^{N_{risk}} \sum_{j=1}^{N_{risk}} \tilde{t}_{I^{'}_{(0,k;1,j)}\to S_{i}}G_{ij}\frac{\rho_{I^{'}_{(0,k;1,j,ART)}}I^{'}_{(0,k;1,j,ART)}}{H_{j}} +\sum_{j=1}^{N_{risk}} \sum_{k=1}^{N_{risk}} \tilde{t}_{I^{'}_{(1,k,ART;0,j)}\to S_{i}}G_{ik}\frac{\rho_{I^{'}_{(1,k,ART;0,j)}}I^{'}_{(1,k,ART;0,j)}}{H_{k}} \\ +\sum_{k=1}^{N_{risk}} \sum_{j=1}^{N_{risk}} \tilde{t}_{I^{'}_{(1,k,ART;1,j)}\to S_{i}}G_{ij}\frac{\rho_{I^{'}_{(1,k,ART;1,j)}}I^{'}_{(1,k,ART;1,j)}}{H_{j}} +\sum_{j=1}^{N_{risk}} \sum_{k=1}^{N_{risk}} \tilde{t}_{I^{'}_{(1,k;1,j,ART)}\to S_{i}}G_{ik}\frac{\rho_{I^{'}_{(1,k;1,j,ART)}}I^{'}_{(1,k;1,j,ART)}}{H_{k}} \\ +\sum_{k=1}^{N_{risk}} \sum_{j=1}^{N_{risk}} \tilde{t}_{I^{'}_{(1,k,ART;1,j,ART)}\to S_{i}}G_{ij}\frac{\rho_{I^{'}_{(1,k,ART;1,j,ART)}}I^{'}_{(1,k,ART;1,j,ART)}}{H_{j}} \left.+\sum_{j=1}^{N_{risk}} \sum_{k=1}^{N_{risk}} \tilde{t}_{I_{(1,k,ART;1,j,ART)}^{'}\to S(i)}G_{ik}\frac{\rho_{I_{(1,k;1,j,ART)}^{'}}I^{'}_{(1,k,ART;1,j,ART)}}{H_{k}}\right) \end{matrix}$$

where $\rho_{X_{i}}$ describes the effective new partner acquisition rate for any population variable $X_{i}$ ($S_{i}$, $I_{i}$, $S_{(i,j)}^{'}$, $I_{(1,i;0,j)}^{'}$, $I_{(0,i;1,j)}^{'}$, $I_{(1,i;1,j)}^{'}$, $I_{(1,i,ART;0,j)}^{'}$, $I_{(0,i;1,j,ART)}^{'}$, $I_{(1,i,ART;1,j)}^{'}$, $I_{(1,i;1,j,ART)}^{'}$, or $I_{(1,i,ART;1,j,ART)}^{'}$; further discussion in section below).

The parameters $t_{I_{j}\to S_{i}}$ and $t_{I_{j,ART}\to S_{i}}$ (HIV transmission within a casual partnership between an infected single individual and an uninfected single individual) and $\tilde{t}_{I_{j}^{'}\to S_{i}}$ and $\tilde{t}_{I_{j,ART}^{'}\to S_{i}}$ (HIV transmission within a casual partnership between an infected married individual and an uninfected single individual) define HIV transmission probability per partnership between a member of the susceptible population and a member of the HIV infected population (in absence or presence of ART). It is expressed in terms of HIV transmission probability per coital act per HIV stage in this partnership ($p_{I_{j}\to S_{i}}^{HIV}$ or $p_{I_{j,ART}\to S_{i}}^{HIV}$), the frequency of coital acts per unit time in this partnership ($n_{I_{j}\leftrightarrow S_{i}}$ or $n_{I_{j,ART}\leftrightarrow S_{i}}$), and the duration ($\tau_{I_{j}\leftrightarrow S_{i}}$, $\tau_{I_{j,ART}\leftrightarrow S_{i}}$) of this partnership:

$$t_{I_{j}\to S_{i}}=\tilde{t}_{I_{j}^{'}\to S_{i}}=1-\left(1-p_{I_{j}\to S_{i}}^{HIV}\right)^{n_{I_{j}\leftrightarrow S_{i}}\times\tau_{I_{j}\leftrightarrow S_{i}}}$$

$$t_{I_{j,ART}\to S_{i}}=\tilde{t}_{I_{j,ART}^{'}\to S_{i}}=1-\left(1-p_{I_{j,ART}\to S_{i}}^{HIV}\right)^{n_{I_{j,ART}\leftrightarrow S_{i}}\times\tau_{I_{j,ART}\leftrightarrow S_{i}}}$$

where $n_{I_{j,ART}\leftrightarrow S_{i}}=n_{I_{j}\leftrightarrow S_{i}}$, $\tau_{I_{j,ART}\leftrightarrow S_{i}}=\tau_{I_{j}\leftrightarrow S_{i}}$, and $p_{I_{j,ART}\to S_{i}}^{HIV}=r\times p_{I_{j,}\to S_{i}}^{HIV}$.

The mixing among the different risk groups was dictated by the mixing matrix $G_{i,m}$. This matrix provides the probability that an individual in the *i*- risk group would choose a partner from the *j*-risk group. It is given by:

$$G_{ij}=e\delta_{ij}+(1-e)\frac{H_{j}}{\sum_{j=1}^{N_{risk}} H_{j}}$$

where

$$\begin{matrix} H_{j}=\rho_{S_{j}}S_{j}+\sum_{h=1}^{N_{risk}} \left[\rho_{S_{(h,j)}^{'}}S_{(h,j)}^{'}+\rho_{S_{(j,h)}^{'}}S_{(j,h)}^{'}\right]+\rho_{I_{j}}I_{j} \\ +\sum_{h=1}^{N_{risk}} \left[\rho_{I_{(0,h,1,j)}^{'}}I_{(0,h,1,j)}^{'}+\rho_{I_{(0,j,1,h)}^{'}}I_{(0,j;1,h)}^{'}\right] +\sum_{h=1}^{N_{risk}} \left[\rho_{I_{(0,h,1,j)}^{'}}I_{(1,h;0,j)}^{'}+\rho_{I_{(0,j,1,h)}^{'}}I_{(1,j;0,h)}^{'}\right] \\ +\sum_{h=1}^{N_{risk}} \left[\rho_{I_{(1,h,1,j)}^{'}}I_{(1,h,1,j)}^{'}+\rho_{I_{(1,j,1,h)}^{'}}I_{(1,j;1,h)}^{'}\right]+\rho_{I_{j,ART}}I_{j,ART} \\ +\sum_{h=1}^{N_{risk}} \left[\rho_{I_{(0,h,1,j,ART)}^{'}}I_{(0,h;1,j,ART)}^{'}+\rho_{I_{(0,j,1,h,ART)}^{'}}I_{(0,j;1,h,ART)}^{'}\right] +\sum_{h=1}^{N_{risk}} \left[\rho_{I_{(0,h,1,j,ART)}^{'}}I_{(1,h,ART;0,j)}^{'}+\rho_{I_{(0,j,1,h,ART)}^{'}}I_{(1,j,ART;0,h)}^{'}\right] \\ +\sum_{h=1}^{N_{risk}} \left[\rho_{I_{(1,h,ART;1,j,ART)}^{'}}I_{(1,h,ART;1,j,ART)}^{'}+\rho_{_{I(1,j,ART;1,h,ART)}^{'}}I_{(1,j,ART;1,h,ART)}^{'}\right] \\ +\sum_{h=1}^{N_{risk}} \left[\rho_{I_{(1,h,ART,1,j)}^{'}}I_{(1,h,ART;1,j)}^{'}+\rho_{I_{(1,j,1,h,ART)}^{'}}I_{(1,j,ART;1,h)}^{'}\right]+\sum_{h=1}^{N_{risk}} \left[\rho_{I_{(1,h,1,j,ART)}^{'}}I_{(1,h;1,j,ART)}^{'}+\rho_{I_{(1,j,1,h,ART)}^{'}}I_{(1,j;1,h,ART)}^{'}\right] \end{matrix}$$

$H_{j}$ is the total number of partnerships (per year) made by the total population in risk group *j*. Here, $\delta_{i,m}$ is again the Kronecker delta and the parameter $e\in\left[0,1\right]$ measures the degree of assortativeness in the mixing. At the extreme, $e=0$, the mixing is proportionate (choosing partners with no preferential bias based on the kind of risk group) while at the other extreme, $e=1$, the mixing is fully assortative as individuals choose partners only from within their own risk group [[2](#_ENREF_2)].

- 1. **Sexual risk behavior in the population**

*2.1 Distribution of sexual risk behavior*

The population in reproductive age was stratified into a number of risk groups. In absence of direct empirical data to inform the exact distribution of sexual risk behavior in a given population, we assumed that the proportion of the population initially in each risk group $i$ follows a gamma distribution. This distribution was informed by the degree distribution of the number of sexual partners in sub-Saharan Africa [[3](#_ENREF_3)]. The gamma distribution of the population fraction by risk group is given by:

$$p(i)=\frac{1}{b^{a}\Gamma(a)}i^{a-1}e^{\frac{-i}{b}}$$

Here, $a$ is the shape parameter determined through normalization of the distribution, and $b$ is the scale parameter in the gamma distribution.

*2.2 The effective new sexual partner change rate*

The $\rho_{X_{i}}$ parameter describes the number of new sexual partners an individual in a specified risk group acquires, but also effectively other factors that enhance the risk of exposure to the infection such as concurrency and clustering within sexual networks [[4-9](#_ENREF_4)], and variability in sexual risk behavior in the population [[10](#_ENREF_10)]. Since the exact nature of sexual risk behavior is not well-understood and varies within and across communities [[11](#_ENREF_11), [12](#_ENREF_12)]. $\rho_{X_{i}}$ is effectively a summary measure of the population-specific level of sexual risk behavior, and captures the distribution and strength of the risk of exposure to HIV infection. We assumed that the form of the $\rho_{X_{i}}$ distribution across different risk groups follows a power law function as [[13](#_ENREF_13)]:

$$\rho_{X_{i}}\text{ }=Ci^{\sigma}$$

Here $\sigma$ is the exponent in the power-law function that determines the level of variability in the effective sexual partner change rate, and $C$ is an overall constant determined by the average risk behavior.

*2.3 Temporal variation in sexual risk behavior*

Given the evidence for rapidly declining HIV incidence in sub-Saharan Africa [[14-16](#_ENREF_14)], we incorporated in our model temporal changes in sexual risk behavior. We parameterized the temporal variation (time dependence of $\rho_{X_{i}}$) through a Wood-Saxon function [[17](#_ENREF_17), [18](#_ENREF_18)] as described in Awad et al [[13](#_ENREF_13)]. Briefly, through the Wood-Saxon parameterization, $\rho_{X_{i}}(t)$ is given by:

$\rho_{X_{i}}(t)=\rho_{X_{i}}\left(1+\frac{Z}{1+\exp\left[\left(t-\xi_{Turning}\right)/\xi_{Duration}\right]}\right)$ .

Here, $\rho_{X_{i}}$ is the asymptotic value of $\rho_{X_{i}}(t)$ that describes the level of risk behavior well after the transition. $\xi_{Duration}$ describes the transition duration parameter, with the actual duration of the transition given by $\tau=4.4\xi_{Duration}$ (where the effective partner change rate falls from 90% to 10% of the difference between initial and final levels of sexual risk behavior) [[17](#_ENREF_17)]. Meanwhile, $\xi_{Turning}$ is the turning point year at which the effective partner change rate crosses half the way towards its asymptotic value of $\rho_{X_{i}}$.

The level of sexual risk behavior changes during the transition from $\rho_{X_{i}}\left(1+Z\right)$ before the transition to $\rho_{X_{i}}$ after the transition. Accordingly, the reduction in the level of sexual risk behavior is given by .

- 1. **Model parameters**

We parameterized our model using nationally-representative empirical data on HIV epidemiology and natural history [[15](#_ENREF_15), [19-21](#_ENREF_19)], as listed in S1 Table along with their references.

- 1. **Model fits**

The model was fitted using a nonlinear least-square fitting method that incorporates an algorithm specifically designed to find the best fit for non-linear functions through an iterative process. This technique, implemented in MATLAB [[22](#_ENREF_22)], minimizes the sum of squares between all data points and the nonlinear model, using the Nelder-Mead simplex algorithm as described in Lagarias et al [[23](#_ENREF_23)]. The fitting procedure is a direct search method that attempts to minimize the error between two sets of data, in this case the empirical and the predicted measures by adjusting seven model parameters until specific convergence criteria are met. Model fits were conducted for the HIV prevalence time-series data and seven key statistics, relating to sero-discordancy, whose empirical values were derived from different rounds of the country-specific DHS.

The parameters that were adjusted to provide the best fit for each country include: country-specific annual risk of HIV transmission from the infected to the uninfected partner in a stable HIV sero-discordant couple () [[24](#_ENREF_24)]—that is HIV incidence rate within stable HIV sero-discordant couples, the stable couple formation rate ($\omega$), the size of the epidemic at the year HIV was seeded into the simulations (1970), the average level of sexual risk behavior in the population (*C*), the scale of the reduction in average level of sexual risk behavior in the population in recent years (the reduction in effective partnership change rate;$Z$), the duration of the sexual risk transition (the time needed for the effective partnership change rate to fall from 90% to 10% of the difference between initial and final levels of sexual risk behavior [[17](#_ENREF_17), [25](#_ENREF_25)]; $\xi_{Duration}$), and the turning-point year of the transition (inflexion point exactly halfway through the transition; $\xi_{Turning}$). The country-specific values for these parameters can be found in S2 Table.

**References**

1. Chemaitelly H, Awad SF, Abu-Raddad LJ: **The risk of HIV transmission within HIV-1 sero-discordant couples appears to vary across sub-Saharan Africa**. *Epidemics* 2014, **6**:1-9.

2. Garnett GP, Anderson RM: **Factors controlling the spread of HIV in heterosexual communities in developing countries: patterns of mixing between different age and sexual activity classes**. *Philos Trans R Soc Lond B Biol Sci* 1993, **342**(1300):137-159.

3. Omori R, Chemaitelly H, Abu-Raddad LJ: **Dynamics of non-cohabiting sex partnering in sub-Saharan Africa: a modelling study with implications for HIV transmission**. *Sex Transm Infect* 2015, **91**(6):451-457.

4. Kretzschmar M, Morris M: **Measures of concurrency in networks and the spread of infectious disease**. *Mathematical Biosciences* 1996, **133**(2):165-195.

5. Morris M: **Sexual networks and HIV**. *Aids* 1997, **11**:S209-S216.

6. Watts CH, May RM: **The influence of concurrent partnerships on the dynamics of HIV/AIDS**. *Math Biosci* 1992, **108**(1):89-104.

7. Kretzschmar M, Morris M: **Measures of concurrency in networks and the spread of infectious disease**. *Math Biosci* 1996, **133**(2):165-195.

8. Morris M: **Sexual networks and HIV**. *AIDS* 1997, **11 Suppl A**:S209-216.

9. Abu-Raddad LJ, Longini IM, Jr.: **No HIV stage is dominant in driving the HIV epidemic in sub-Saharan Africa**. *AIDS* 2008, **22**(9):1055-1061.

10. May RM, Anderson RM: **The Transmission Dynamics of Human Immunodeficiency Virus (Hiv)**. *Philosophical Transactions of the Royal Society of London Series B-Biological Sciences* 1988, **321**(1207):565-607.

11. Ferry B, Carael M, Buve A, Auvert B, Laourou M, Kanhonou L, de Loenzien M, Akam E, Chege J, Kaona F: **Comparison of key parameters of sexual behaviour in four African urban populations with different levels of HIV infection**. *AIDS* 2001, **15 Suppl 4**:S41-50.

12. Lagarde E, Auvert B, Carael M, Laourou M, Ferry B, Akam E, Sukwa T, Morison L, Maury B, Chege J *et al*: **Concurrent sexual partnerships and HIV prevalence in five urban communities of sub-Saharan Africa**. *Aids* 2001, **15**(7):877-884.

13. Awad SF, Abu-Raddad LJ: **Could there have been substantial declines in sexual risk behavior across sub-Saharan Africa in the mid-1990s?** *Epidemics* 2014, **8**:9-17.

14. UNAIDS/WHO: **AIDS epidemic update 2010: UNAIDS fact sheet** In*.*; 2010. Available: <http://www.unaids.org/documents/20101123_FS_SSA_em_en.pdf>.

15. UNAIDS: **UNAIDS Report on the Global AIDS Epidemic 2010**. In*.*; 2010.

16. Mahboob A, Haroon TS, Iqbal Z, Saleemi MA, Munir A: **Prevalence of hepatitis B surface antigen carrier state in patients with lichen planus--report of 200 cases from Lahore, Pakistan**. *J Ayub Med Coll Abbottabad* 2007, **19**(4):68-70.

17. Velicia FJF: **On the moments of a Wood Saxon beta distribution**. *Journal of Physics A: Mathematical and General* 1987.

18. Woods RD, Saxon DS: **Diffuse Surface Optical Model for Nucleon-Nuclei Scattering**. *Physical Review* 1954, **95**(2):577-578.

19. UNAIDS: **UNAIDS Reference Group on Estimates, Modelling and Projections**. 2007.

20. Morgan D, Whitworth J: **The natural history of HIV-1 infection in Africa**. *Nat Med* 2001, **7**(2):143-145.

21. Baeten JM, Richardson BA, Lavreys L, Rakwar JP, Mandaliya K, Bwayo JJ, Kreiss JK: **Female-to-male infectivity of HIV-1 among circumcised and uncircumcised Kenyan men**. *J Infect Dis* 2005, **191**(4):546-553.

22. MATLAB®: **The Language of Technical Computing**. In*.*, 8.1.0.604 (R2013a) edn: The MathWorks, Inc.; 2013.

23. Lagarias JC, J. A. Reeds, M. H. Wright,and P. E. Wright: **Convergence Properties of the Nelder-MeadSimplex Method in Low Dimensions**. *SIAM Journal of Optimization* 1998, **9**(1):112-147.

24. Awad SF, Chemaitelly H, Abu-Raddad LJ: **Estimating the annual risk of HIV transmission within HIV sero-discordant couples in sub-Saharan Africa**. *Int J Infect Dis* 2017.

25. Woods RD, Saxon DS: **Diffuse Surface Optical Model for Nucleon-Nuclei Scattering**. *Physical Review* 1954, **95 (2)** 577-578.
