# Supplementary material for: Temporal evolution of HIV sero-discordancy patterns among stable couples in sub-Saharan Africa
Source: PLoS One. 2018 Apr 30;13(4):e0196613. doi: 10.1371/journal.pone.0196613 (PMC5927442; doi:10.1371/journal.pone.0196613)
Supplement: S1 Table — (DOCX) [file pone.0196613.s002.docx]

**Table S1** Model assumptions in terms of parameter values. All of these parameters are independent from the seven fitted parameters in our study.

| **Assumption** | **Parameter value** | **Sources** |
| --- | --- | --- |
| Duration of the sexual activity lifespan ($1/\mu$) | 35 years | [1-3] |
| Duration of HIV infection in absence of ART ($1/\alpha$) | 11 years | [1, 4-6] |
| Duration of HIV infection with ART ($1/\alpha_{ART}$) | 33 years | [7, 8] |
| HIV transmission probability per coital act in absence of ART ($p_{I_{j}\to S_{i}}^{HIV}$) estimated as weighted average of the probability by HIV stage | 0.0016 | [1, 9-11] |
| Reduction in HIV transmission probability per coital act due to ART ($r$) | 96% | [12, 13] |
| Frequency of coital acts per month ($n_{I_{j}\leftrightarrow S_{i}}$and $n_{I_{j,ART}\leftrightarrow S_{i}}$) | 11.0 per month | [11] |
| Duration of stable sexual partnerships ($d=1/\gamma$)  *Niger*  *Mali*  *Tanzania*  *Kenya*  *Zimbabwe*  *Lesotho* | 13.40  12.97  10.25  10.85  9.95  11.07 | Derived from DHS data [14, 15] |
| Duration of casual sexual partnerships ($\tau_{I_{j}\leftrightarrow S_{i}}$ and $\tau_{I_{j,ART}\leftrightarrow S_{i}}$) | 6 months | Representative value |
| Degree of assortativeness ($e$) | 0.3 | [1] |
| The shape parameter in the gamma distribution of the population across the risk groups ($a$) | 1.1 | Derived value to fit HIV epidemic tractecjories [16] |
| The scale parameter in the gamma distribution of the population across the risk groups ($b$) | 1.1 | Derived value to fit HIV epidemic tractecjories [16] |
| The exponent parameter in the power law function of the distribution of sexual risk behavior ($\sigma$) | 1.7 | [16] |
| Country-specific ART coverage by 2014 among all individuals living with HIV (to parametrize ART treatment rate: $\eta$)  *Niger*  *Mali*  *Tanzania*  *Kenya*  *Zimbabwe*  *Lesotho* | 22%  24%  55%  43%  51%  35% | [17] |

ART: antiretroviral therapy; DHS: Demographic and Health Surveys

**References**

1. Abu-Raddad LJ, Longini IM, Jr. No HIV stage is dominant in driving the HIV epidemic in sub-Saharan Africa. AIDS. 2008;22(9):1055-61. Epub 2008/06/04. doi: 10.1097/QAD.0b013e3282f8af84. PubMed PMID: 18520349.

2. Buve A, Carael M, Hayes RJ, Auvert B, Ferry B, Robinson NJ, et al. Multicentre study on factors determining differences in rate of spread of HIV in sub-Saharan Africa: methods and prevalence of HIV infection. AIDS. 2001;15 Suppl 4:S5-14. Epub 2001/11/01. PubMed PMID: 11686465.

3. UNAIDS. UNAIDS Report on the Global AIDS Epidemic 2010 2010. Available from: <http://www.unaids.org/globalreport/Global_report.htm>.

4. UNAIDS. UNAIDS Reference Group on Estimates, Modelling and Projections. 2007.

5. Morgan D, Whitworth J. The natural history of HIV-1 infection in Africa. Nat Med. 2001;7(2):143-5. PubMed PMID: 11175832.

6. Baeten JM, Richardson BA, Lavreys L, Rakwar JP, Mandaliya K, Bwayo JJ, et al. Female-to-male infectivity of HIV-1 among circumcised and uncircumcised Kenyan men. J Infect Dis. 2005;191(4):546-53. PubMed PMID: 15655778.

7. Stover J, Johnson P, Hallett T, Marston M, Becquet R, Timaeus IM. The Spectrum projection package: improvements in estimating incidence by age and sex, mother-to-child transmission, HIV progression in children and double orphans. Sex Transm Infect. 2010;86 Suppl 2:ii16-21. Epub 2010/12/09. doi: 86/Suppl_2/ii16 [pii]

10.1136/sti.2010.044222. PubMed PMID: 21106510.

8. Granich R, Gupta S, Hersh B, Williams B, Montaner J, Young B, et al. Trends in AIDS Deaths, New Infections and ART Coverage in the Top 30 Countries with the Highest AIDS Mortality Burden; 1990-2013. PLoS One. 2015;10(7):e0131353. doi: 10.1371/journal.pone.0131353. PubMed PMID: 26147987; PubMed Central PMCID: PMCPMC4493077.

9. Pinkerton SD. Probability of HIV transmission during acute infection in Rakai, Uganda. AIDS Behav. 2008;12(5):677-84. Epub 2007/12/08. doi: 10.1007/s10461-007-9329-1. PubMed PMID: 18064559.

10. Hollingsworth TD, Anderson RM, Fraser C. HIV-1 transmission, by stage of infection. J Infect Dis. 2008;198(5):687-93. Epub 2008/07/30. doi: 10.1086/590501. PubMed PMID: 18662132.

11. Wawer MJ, Gray RH, Sewankambo NK, Serwadda D, Li X, Laeyendecker O, et al. Rates of HIV-1 transmission per coital act, by stage of HIV-1 infection, in Rakai, Uganda. J Infect Dis. 2005;191(9):1403-9. Epub 2005/04/06. doi: 10.1086/429411. PubMed PMID: 15809897.

12. Cohen MS, Chen YQ, McCauley M, Gamble T, Hosseinipour MC, Kumarasamy N, et al. Prevention of HIV-1 infection with early antiretroviral therapy. N Engl J Med. 2011;365(6):493-505. doi: 10.1056/NEJMoa1105243. PubMed PMID: 21767103; PubMed Central PMCID: PMCPMC3200068.

13. Donnell D, Baeten JM, Kiarie J, Thomas KK, Stevens W, Cohen CR, et al. Heterosexual HIV-1 transmission after initiation of antiretroviral therapy: a prospective cohort analysis. Lancet. 2010;375(9731):2092-8. doi: 10.1016/S0140-6736(10)60705-2. PubMed PMID: 20537376; PubMed Central PMCID: PMC2922041.

14. MEASURE DHS. Demographic and health surveys Calverton: ICF Macro; 2012 [updated 2012; cited 2010 May 19,]. Available from: <http://www.measuredhs.com/data/available-datasets.cfm>.

15. Chemaitelly H, Awad SF, Abu-Raddad LJ. The risk of HIV transmission within HIV-1 sero-discordant couples appears to vary across sub-Saharan Africa. Epidemics. 2014;6:1-9. Epub 2014/03/07. doi: 10.1016/j.epidem.2013.11.001. PubMed PMID: 24593916.

16. Awad SF, Abu-Raddad LJ. Could there have been substantial declines in sexual risk behavior across sub-Saharan Africa in the mid-1990s? Epidemics. 2014;8:9-17. doi: 10.1016/j.epidem.2014.06.001. PubMed PMID: 25240899.

17. UNAIDS. AIDSinfo. Coverage of people recieving ART (available at: <http://aidsinfo.unaids.org/>. Accessed April, 2016) 2015.
