# Supplementary material for: Temporal evolution of HIV sero-discordancy patterns among stable couples in sub-Saharan Africa
Source: PLoS One. 2018 Apr 30;13(4):e0196613. doi: 10.1371/journal.pone.0196613 (PMC5927442; doi:10.1371/journal.pone.0196613)
Supplement: S2 Table — (DOCX) [file pone.0196613.s003.docx]

**S2 Table.** Estimated parameter values for the six countries included in our study. Countries are shown in order of increasing HIV prevalence.

|  | **Niger** | **Mali** | **Tanzania** | **Kenya** | **Zimbabwe** | **Lesotho** |
| --- | --- | --- | --- | --- | --- | --- |
| Stable couple formation rate ($\omega$; [*per year*]) | 0.23 | 0.60 | 0.24 | 0.20 | 0.24 | 0.17 |
| Mean annual risk of HIV transmission from the infected to the uninfected partner in a stable HIV sero-discordant couple (; [*per 100 person-year*]) | 18.55 | 12.62 | 11.24 | 12.30 | 27.08 | 30.71 |
| Average level parameter of sexual risk behavior in the population (*C*) | 0.01 | 0.02 | 0.06 | 0.03 | 0.04 | 0.15 |
| Scale of the reduction in the average level of sexual risk behavior in the population (*$Z$*) | 10.00 | 8.39 | 2.19 | 6.40 | 7.78 | 1.06 |
| Duration of the sexual risk transition ($\xi_{Duration}$; [*years*]) | 27.90 | 1.33 | 1.38 | 26.49 | 26.32 | 29.70 |
| Turning-point year of the sexual risk transition ($\xi_{Turning}$; [*year*]) | 1994 | 1990 | 1992 | 1991 | 1993 | 1994 |
