# Supplementary material for: Temporal evolution of HIV sero-discordancy patterns among stable couples in sub-Saharan Africa
Source: PLoS One. 2018 Apr 30;13(4):e0196613. doi: 10.1371/journal.pone.0196613 (PMC5927442; doi:10.1371/journal.pone.0196613)
Supplement: S3 Table — (DOCX) [file pone.0196613.s004.docx]

**S3 Table.** Empirical values and 95 confidence interval (CI) of HIV prevalence and the key HIV sero-discordancy measures for six representative countries in sub-Saharan Africa as estimated from the Demographic Health Surveys (DHS) databases. Countries are shown in order of increasing HIV prevalence.

| Country | Year(s) | $P$ (95% CI)  [%] | $P_{all}$ (95% CI)  [%] | $P_{discord}$ (95% CI)  [%] | $I_{pos}$ (95% CI)  [%] | $I_{all}$ (95% CI)  [%] | $P_{pos}$ (95% CI)  [%] | $P_{couple}$ (95% CI)  [%] |
| --- | --- | --- | --- | --- | --- | --- | --- | --- |
| Niger | 2012 | 0.36 (0.26, 0.51) | 0.27 (0.11, 0.57) | 61.39 (35.43, 84.80) | 36.06 (11.82, 61.62) | 0.21 (0.11, 0.39) | 0.44 (0.25, 0.77) | 79.20 (79.16, 79.23) |
|  | 2006 | 0.68 (0.51, 0.91) | 0.97 (0.60, 1.50) | 84.90 (67.99, 93.81) | 50.13 (31.95, 71.33) | 0.74 (0.49, 1.05) | 1.14 (0.75, 1.74) | 76.31 (76.27, 76.34) |
| Mali | 2012-13 | 1.14 (0.91, 1.42) | 1.69 (1.24, 2.27) | 89.29 (76.90, 96.45) | 69.77 (56.39, 82.02) | 1.29 (1.00, 1.64) | 1.89 (1.39, 2.57) | 76.11 (76.08, 76.14) |
|  | 2006 | 1.20 (0.96, 1.50) | 1.16 (0.79, 1.68) | 76.65 (59.86, 89.58) | 48.11 (32.89, 63.05) | 0.87 (0.63, 1.17) | 1.51 (1.05, 2.18) | 74.92 (74.88, 74.96) |
|  | 2004 | 5.35 (4.91, 5.83) | 5.08 (4.15, 6.12) | 68.31 (60.68, 75.78) | 32.89 (26.48, 40.03) | 3.00 (1.14, 1.91) | 7.44 (6.31, 8.74) | 58.99 (58.96, 59.02) |
| Tanzania | 2012 | 5.12 (4.72, 5.55) | 4.65 (3.97, 5.41) | 66.45 (60.05, 72.83) | 31.84 (26.80, 37.29) | 2.70 (2.33, 3.12) | 7.00 (6.02, 8.12) | 58.02 (58.00, 58.03) |
|  | 2007-08 | 5.73 (5.27, 6.23) | 6.42 (5.53, 7.38) | 73.12 (66.01, 79.37) | 37.00 (31.70, 42.67) | 3.76 (3.27, 4.28) | 8.78 (7.51, 10.25) | 58.55 (58.52, 58.57) |
|  | 2003-04 | 7.03 (6.49, 7.62) | 7.85 (6.75, 9.03) | 75.20 (68.54, 80.73) | 37.50 (31.77, 43.23) | 4.58 (3.98, 5.23) | 10.44 (9.04, 12.03) | 58.33 (58.31, 58.35) |
| Kenya | 2008-09 | 6.36 (5.60, 7.22) | 5.95 (4.69, 7.42) | 66.07 (56.76, 74.16) | 28.27 (21.28, 36.36) | 3.26 (2.60, 4.04) | 9.01 (7.23, 11.16) | 54.86 (54.84, 54.88) |
|  | 2003 | 6.73 (6.07, 7.46) | 7.41 (5.90, 9.11) | 66.99 (57.91, 75.67) | 32.90 (25.48, 40.58) | 4.11 (3.31, 5.03) | 11.06 (9.14, 13.32) | 55.42 (55.40, 55.45) |
|  | 2007 | 14.21 (13.50, 14.96) | 11.03 (9.79, 12.40) | 58.49 (53.77, 62.93) | 26.18 (22.78, 29.89) | 6.47 (5.78, 7.23) | 18.86 (17.18, 20.67) | 58.68 (58.63, 58.73) |
| Zimbabwe | 2015 | 14.12 (13.52, 14.74) | 8.86 (7.87, 9.95) | 45.00 (41.06, 49.02) | 19.30 (16.81, 22.06) | 5.02 (4.48, 5.61) | 19.69 (18.11, 21.38) | 56.67 (56.63, 56.70) |
|  | 2011 | 15.32 (14.69, 15.97) | 11.22 (10.00, 12.57) | 52.39 (48.00, 56.57) | 22.94 (20.02, 26.18) | 6.42 (5.74, 7.20) | 21.43 (19.70, 23.23) | 57.25 (57.21, 57.29) |
|  | 2005-06 | 18.14 (17.35, 18.96) | 13.07 (11.54, 14.67) | 47.27 (42.85, 51.87) | 19.67 (16.88, 22.58) | 6.89 (6.11, 7.77) | 27.65 (25.18, 30.25) | 52.70 (52.65, 52.74) |
| Lesotho | 2009 | 22.97 (21.82, 24.16) | 17.23 (14.72, 20.06) | 47.90 (41.96, 54.08) | 18.61 (15.11, 22.60) | 8.25 (6.96, 9.71) | 35.97 (32.17, 39.94) | 47.90 (47.80, 47.99) |
|  | 2004 | 23.10 (21.74, 24.51) | 13.43 (10.81, 16.59) | 41.06 (33.86, 48.35) | 15.27 (11.31, 19.81) | 6.37 (5.05, 7.98) | 32.71 (28.31, 37.46) | 47.45 (47.34, 47.56) |

$P$: HIV prevalence in the population; $P_{all}$: Proportion of stable HIV sero-discordant couples among all stable couples in the population; $P_{discord}$: Proportion of stable HIV sero-discordant couples among all stable couples with at least one HIV infected individual in the couple; $I_{pos}$: Proportion of all HIV-infected individuals engaged in a stable HIV sero-discordant couple; $I_{all}$: Proportion of individuals in stable HIV sero-discordant couple among the entire reproductive age population; $P_{pos}$: Proportion of couples affected by HIV out of all stable couples; $P_{couples}$: Population prevalence of stable couples
