# Supplementary material for: Temporal evolution of HIV sero-discordancy patterns among stable couples in sub-Saharan Africa
Source: PLoS One. 2018 Apr 30;13(4):e0196613. doi: 10.1371/journal.pone.0196613 (PMC5927442; doi:10.1371/journal.pone.0196613)
Supplement: S2 Fig — (DOCX) [file pone.0196613.s006.docx]

**S2 Fig.** Model predicted HIV prevalence among the population in reproductive age for six representative countries in sub-Saharan Africa. Countries are shown in order of increasing HIV prevalence: (A) Niger, (B) Mali, (C) Tanzania, (D) Kenya, (E) Zimbabwe, and (F) Lesotho. The black lines show model fits for the Demographic and Health Surveys data points (red asterisks) [1] and UNAIDS estimations for 1990-2014 (blue asterisks) [2].


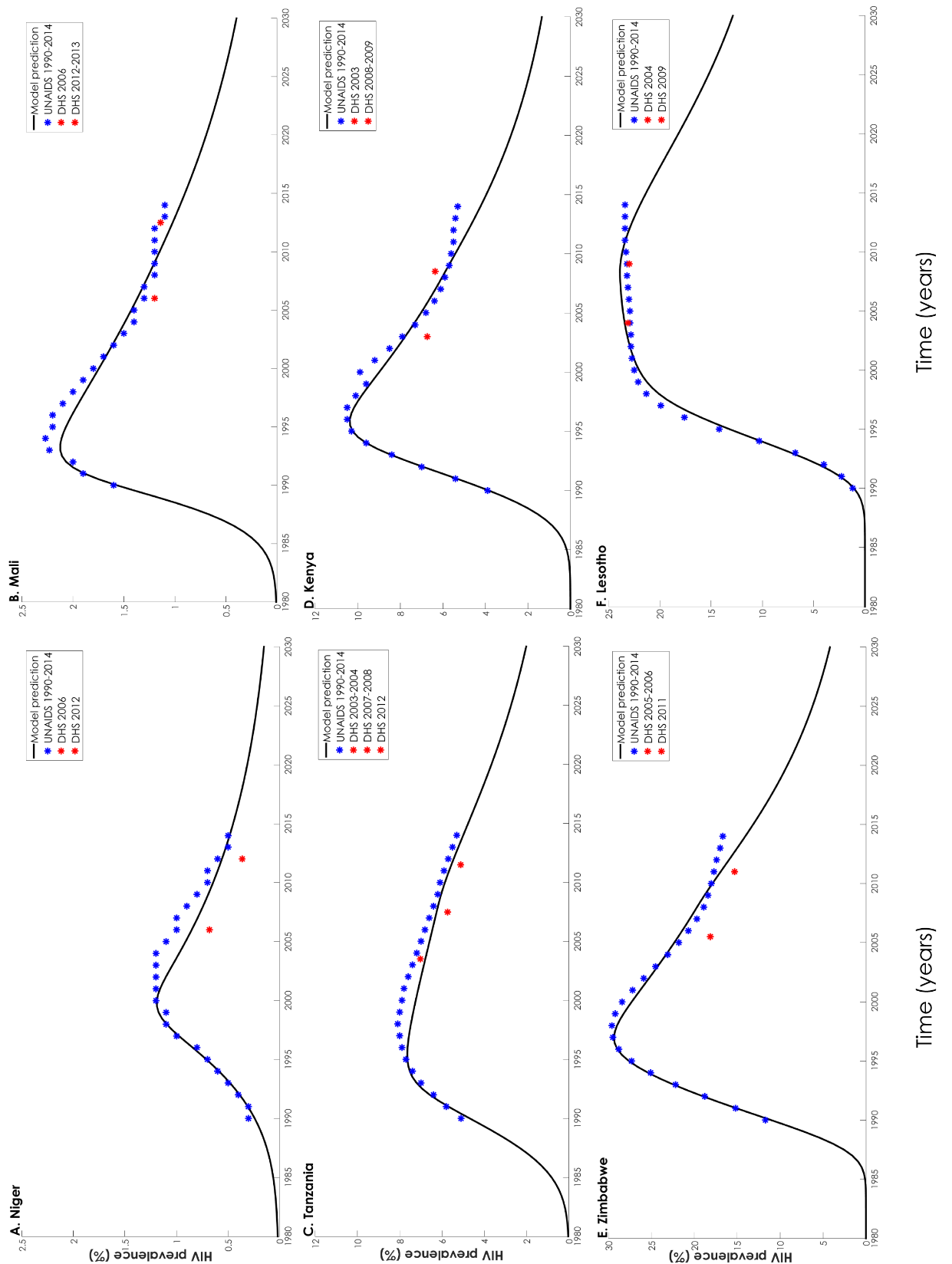


**References**

1. MEASURE DHS. Demographic and health surveys. ICF Macro. Available: <http://www.measuredhs.com/>.

2. UNAIDS. Epidemiological data, HIV estimates 1990-2014. 2014. Available: <http://www.unaids.org/en/dataanalysis/datatools/aidsinfo>.
