# Supplementary material for: Temporal evolution of HIV sero-discordancy patterns among stable couples in sub-Saharan Africa
Source: PLoS One. 2018 Apr 30;13(4):e0196613. doi: 10.1371/journal.pone.0196613 (PMC5927442; doi:10.1371/journal.pone.0196613)
Supplement: S4 Fig — (DOCX) [file pone.0196613.s008.docx]

**S4 Fig.** Model predicted proportion of all HIV-infected individuals in the population that are engaged in stable HIV sero-discordant couples ($I_{pos}$) for six representative countries in sub-Saharan Africa. Countries are shown in order of increasing HIV prevalence: (A) Niger, (B) Mali, (C) Tanzania, (D) Kenya, (E) Zimbabwe, and (F) Lesotho. The black lines show the model predictions while the red asterisks show the Demographic and Health Surveys data points and their 95% confidence intervals [1].


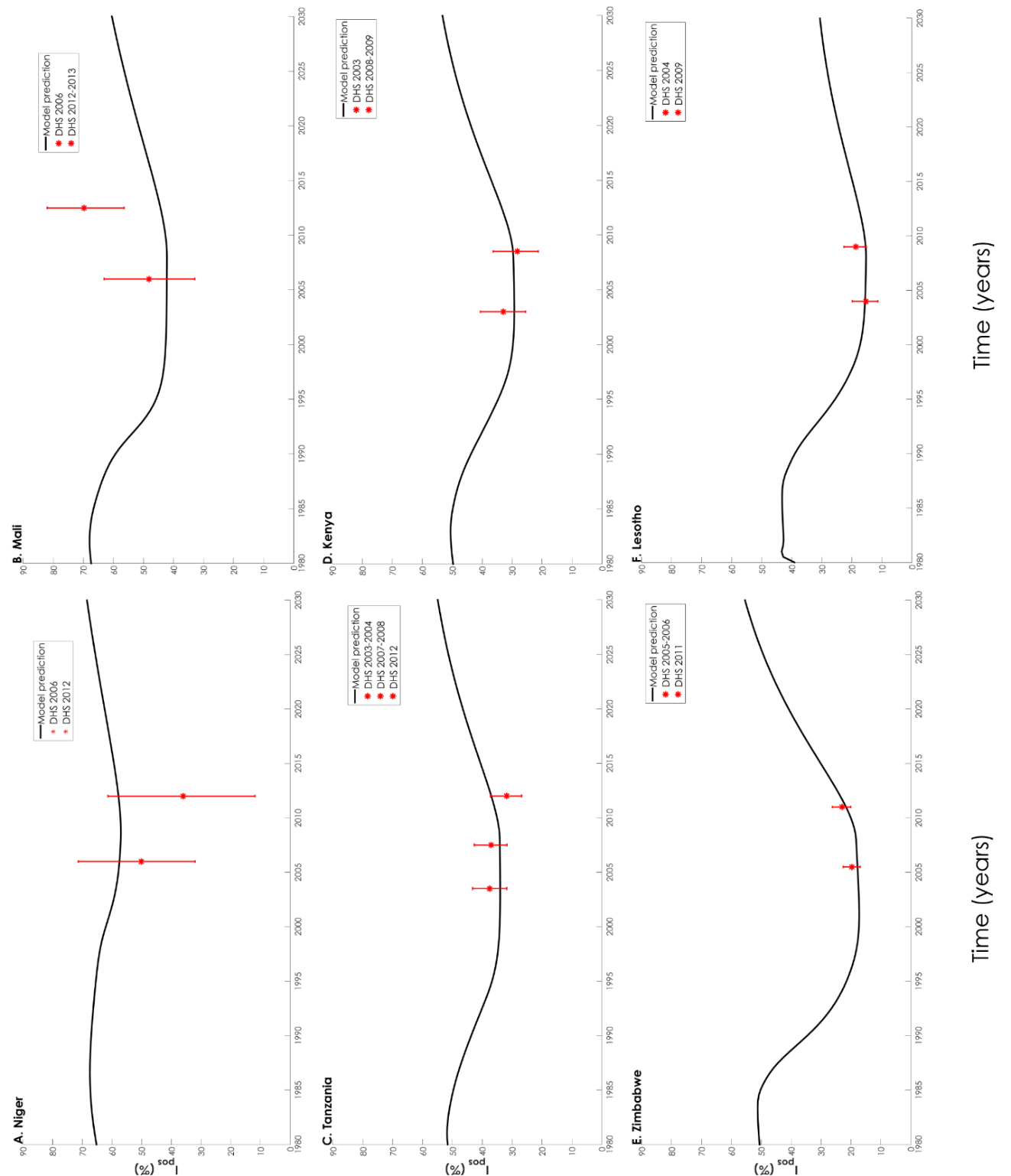


**References**

1. MEASURE DHS. Demographic and health surveys. ICF Macro. Available: <http://www.measuredhs.com/>.
