# Supplementary material for: Temporal evolution of HIV sero-discordancy patterns among stable couples in sub-Saharan Africa
Source: PLoS One. 2018 Apr 30;13(4):e0196613. doi: 10.1371/journal.pone.0196613 (PMC5927442; doi:10.1371/journal.pone.0196613)

**S7 Fig.** Model predicted impact of antiretroviral therapy (ART) scale-up on HIV sero-discordancy. The proportion of stable HIV sero-discordant couples among all stable couples with at least one HIV-infected individual in the couple ($P_{discord}$) with ART scale-up compared to a counter-factual scenario of no-ART scale up, in Kenya as an illustrative example. The black line shows model prediction with current ART scale-up, while the red dashed line shows model prediction without ART scale-up.


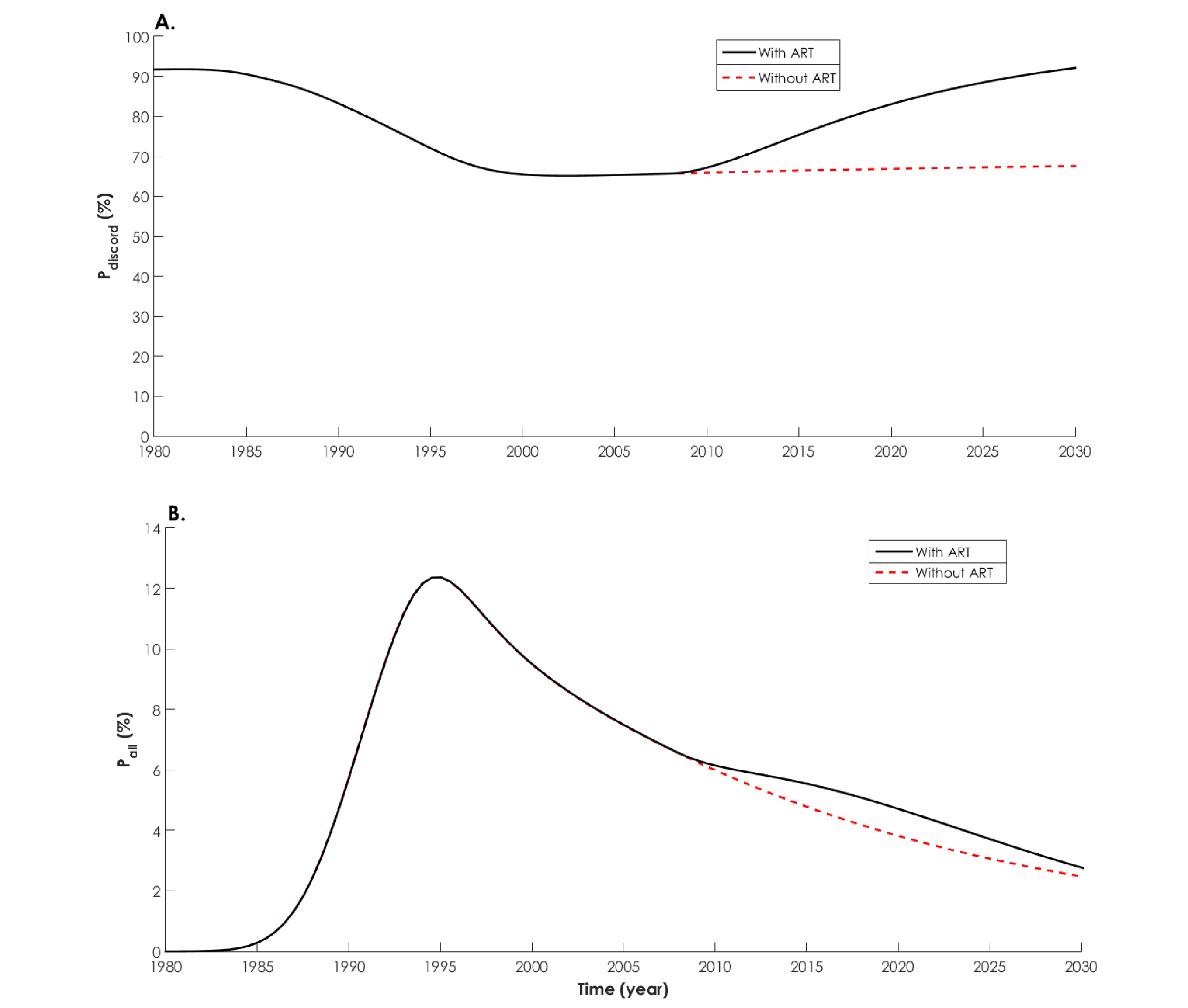

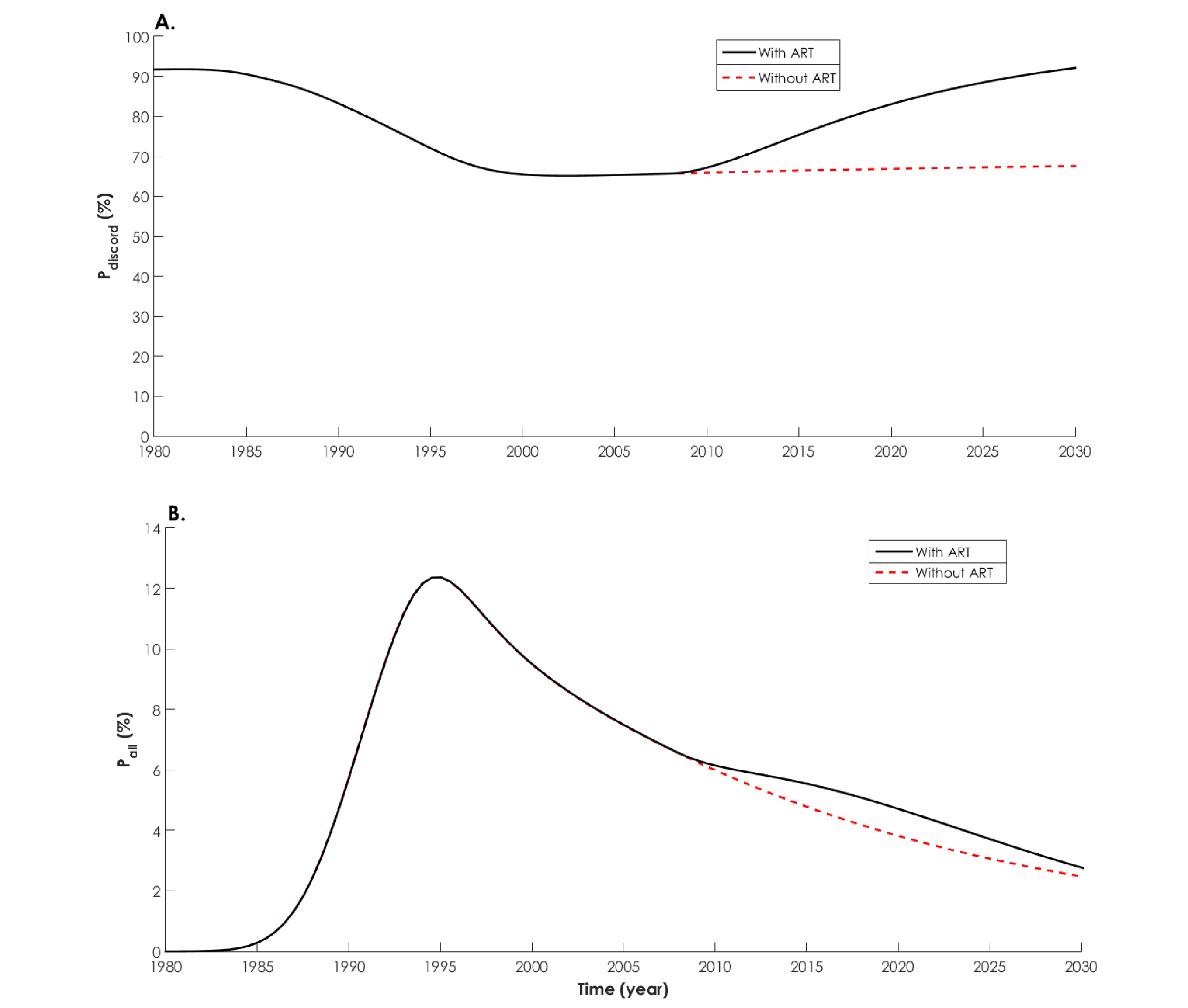

Supplement: S7 Fig — (DOCX) [file pone.0196613.s011.docx]
